# Dimethyl fumarate and extracorporeal photopheresis combination-therapy synergize in inducing specific cell death and long-term remission in cutaneous T cell lymphoma

**DOI:** 10.1038/s41375-024-02479-1

**Published:** 2024-11-23

**Authors:** Özge Ç. Şener, Susanne Melchers, Luisa Tengler, Paul L. Beltzig, Jana D. Albrecht, Deniz Tümen, Karsten Gülow, Jochen S. Utikal, Sergij Goerdt, Tobias Hein, Jan P. Nicolay

**Affiliations:** 1 Department of Dermatology, Venereology and Allergology, University Medical Center Mannheim/ University of Heidelberg, Mannheim, Germany; 2 Skin Cancer Unit, German Cancer Research Center (DKFZ), Heidelberg, Germany; 3 Section of Clinical and Experimental Dermatology, Medical Faculty Mannheim, University of Heidelberg, Mannheim, Germany; 4 Department of Internal Medicine I, Gastroenterology, Hepatology, Endocrinology, Rheumatology and Infectious diseases, University Hospital Regensburg (UKR), Regensburg, Germany; 5 DKFZ-Hector Cancer Institute at the University Medical Center Mannheim, Mannheim, Germany

**Keywords:** T-cell lymphoma, Translational research

## Abstract

Primary cutaneous T cell lymphomas (CTCL) are characterized by high relapse rates to initially highly effective therapies. Combination therapies have proven beneficial, particularly if they incorporate extracorporeal photopheresis (ECP). The NF-κB inhibitor dimethyl fumarate (DMF) has proven a new, effective drug in CTCL in a clinical phase II study. In vitro experiments with patient-derived SS cells and the CTCL cell lines HH, HuT 78, and SeAx revealed a synergistic effect of DMF and ECP on cell death induction in CTCL cells. Furthermore, an additional increase in the capacity to inhibit NF-κB in CTCL was detected for the combination treatment compared to DMF monotherapy. The same synergistic effects could be measured for ROS production *via* decreased Thioredoxin reductase activity and glutathione levels. Consequently, a cell death inhibitor screen indicated that the DMF/ECP combination treatment induces a variety of cell death mechanisms in CTCL. As a first step into clinical translation, 4 patients were already treated with the DMF/ECP combination therapy with an overall response rate of 100% and a time to next treatment in skin and blood of up to 57 months. Therefore, our study introduces the combination treatment of DMF and ECP as a highly effective and long-lasting CTCL therapy.

## Introduction

Primary cutaneous T cell lymphomas (CTCL) are a heterogeneous group of lymphoproliferative malignancies originating in the skin that may subsequently progress to other compartments like the lymph nodes, viscera and the peripheral blood. Mycosis fungoides (MF) and its leukemic variant Sézary Syndrome (SS) represent the most frequently observed CTCL entities [1, 2]. CTCL therapy includes systemic therapy with various medical agents, and extracorporeal photopheresis (ECP) [3, 4]. During ECP, the peripheral blood is UV-sensitized by the administration of 8-Methoxy psoralene (8-MOP) and treated extracorporeally with UVA irradiation [3–5]. ECP is in clinical use for the treatment of CTCL since the 1980s and its efficacy is undisputed, but the exact mode of action of ECP has not yet been comprehensively clarified [5–11]. However, CTCL is characterized by high relapse rates and therapy resistance, even after initially highly effective therapies [12, 13]. Therefore, sequential and combinatory therapy regimes are increasingly administered, and patients particularly benefit from an early inclusion of the ECP in their combinatory therapeutic regimens [14–18]. However, only limited data on the molecular mechanisms behind the clinically observed responses are available.

One reason for clinical and laboratory relapses in CTCL is the malignant cells’ resistance to cell death induction rather than hyperproliferation [19–21]. Recent genomic studies identified the NF-κB, JAK/STAT, PI3K/mTOR, MAPK and T cell receptor signaling pathways to be involved in the pathogenesis of CTCL [22, 23]. Particularly, constitutive NF-κB activation contributes to CTCL cell death resistance. The small molecule NF-κB inhibitor DMF emerged as a promising and well-tolerated CTCL therapeutic in a multicentric clinical phase II study on DMF treatment in relapsed and refractory CTCL patients [24]. In vitro, DMF restores apoptosis sensitivity in CTCL cells, and together with Bcl-2 inhibition synergistically induces cell death in a mouse model in vivo [25, 26]. Translational experiments revealed that DMF appears to inhibit NF-κB activity by interfering with thioredoxin, the major redox regulator of NF-κB transcriptional activity [27]. Besides, inhibition of NF-κB leads to the production of reactive oxygen species (ROS), and the induction of iron-dependent cell death in CTCL [28, 29]. In a clinical phase II study, DMF was particularly effective in the skin compartment in SS patients with a high tumor burden in four out of five patients. Due to its excellent tolerability, DMF has proven an ideal combination therapy partner in CTCL.

In this study, we first investigate the effects of DMF and 8-MOP/UVA mono- and combination therapy on cell death in patient-derived SS cells and CTCL cell lines in vitro and conducted mechanistic experiments on the putative synergistic molecular mechanism. To gain insight into the potential to translate this therapy into clinics, we already treated four CTCL patients with the DMF and ECP combination therapy. In this study, we present the underlying molecular mechanisms from the in vitro DMF and 8-MOP/UVA combination treatment as well as the clinical results from the first four human patients treated with the DMF/ECP combination therapy.

## Materials/subjects and methods

### Reagents and cell lines

Dimethyl Fumarate (DMF), Ferrostatin-1 (Fer-1), Necrostatin-1, Glutathione Monoethyl Ester (GSH) and (±)-6-Hydroxy-2,5,7,8-tetramethylchromane-2-carboxylic acid (Trolox) were purchased from Sigma Aldrich (St. Louis, Missouri, USA). z-VAD-FMK (zVAD) and 8-Methoxypsoralen (8-MOP) were obtained from MedChemExpress (Monmouth Junction, New Jersey, USA). AnnexinV-FITC was purchased from BioLegend (San Diego, California, USA) and 7-aminoactinomycin D (7-AAD) from Tocris (Bristol, UK). Dichlorodihydrofluorescein-diacetate (H_2_DCFDA) was obtained from Invitrogen (Carlsbad, California, USA). Human T lymphocyte cell lines HH, HuT 78, and SeAx cells were cultured in RPMI-1640 medium supplemented with 10% heat-inactivated fetal calf serum (FCS, all Bio&Sell, Nuremberg, Germany). HH and HuT 78 cells were supplied by the American Type Culture Collection (ATCC, Manassas, Virginia, USA) and SeAx cells were kindly provided by Keld Kaltoft from the Aarhus University in Denmark.

### Lymphocyte isolation

SS patient-derived CD4^+^ T cells were separated and cultured as described before [25, 26]. According to Melchers et al., it was assumed that in patients with high tumor load, the biology and functionality of CD4+ cells and Vß-clonal Sézary cells is almost congruent in patients with high tumor load in the blood [30].

### In vitro combination treatment with DMF and 8-MOP/UVA

Cell lines and patient-derived CD4^+^ T cells were treated with 30–50 µM DMF and subjected to 0.5–2 J UVA irradiation using a UVP Ultraviolet Crosslinker CL-100 (Analytik Jena, Jena, Germany) in the presence of 8-MOP both as monotreatment or as combination treatment. DMSO- and untreated samples were prepared as controls. DMF and 8-MOP/UVA dosage was titrated individually (Supplementary Fig. 1); the exact conditions are provided in the Supplementary Table 1.

### Immunoblot

The primary antibodies used are presented in Supplementary Table 2. NuPAGE™Novex™ 10% Bis-Tris Protein Gels (Invitrogen, Carlsbad, California, USA) were run, and transferred to polyvinylidenefluoride membranes (Merck Millipore, Burlington, Massachusetts, USA) that were developed using Luminata Forte Western HRP Substrate (Merck Millipore, Burlington, Massachusetts, USA).

### Immunofluorescence

In brief, immunofluorescent stainings were performed on FFPE-sections after heat-induced antigen retrieval. The primary antibodies are presented in Supplementary Table 4 and were combined with the appropriate fluorophore-conjugated secondary antibodies and 4’,6-diamidino-2-phenylindole (DAPI) as nuclear stain. Pictures were taken with a Keyence BZ-810 microscope (Keyence, Osaka, Japan). The detailed procedures are provided in the supplements.

### ROS generation assay

Intracellular ROS levels were detected using H_2_DCFDA (Thermo Fisher, Waltham, Massachusetts, USA). The cells were treated with DMF and 8-MOP/UVA and stained with the oxidation-sensitive dye H_2_DCFDA (5 μM) for 30 min at 37 °C. ROS production was measured by flow cytometry and quantified according to the mean fluorescence intensity (MFI) [31].

### Flow cytometry

Intracellular staining was performed with the Intracellular Fixation & Permeabilization Buffer Set (Thermo Fisher, Waltham, Massachusetts, USA) according to the manufacturer’s instructions. Cell death was measured by AnnexinV-FITC/7-AAD staining.

### Thioredoxin activity and total glutathione levels

The Thioredoxin Reductase Colorimetric Assay Kit (Cayman, Ann Arbor, Michigan, USA) was used according to manufacturer’s recommendations. Thioredoxin reductase activity was measured at the absorbance of 405 nm. Glutathione levels were measured using the OxiSelect^TM^ Total Glutathione (GSSG/GSH) Assay Kit (Cell Biolabs, San Diego, California, USA) according to manufacturer’s instructions and measured at the absorbance of 405 nm.

### Patients

Four SS patients diagnosed according to WHO-EORTC classification of CTCL and criteria of the International Society of Cutaneous Lymphomas (ISCLC) were treated with the combination therapy of DMF and ECP as compassionate use [2]. For the translational study, peripheral blood samples were collected from five additional SS patients who did not receive the combination therapy. The clinical data of the patients are provided in Supplementary Table 3. Written informed consent was obtained from all patients including publication of clinical photographs. Eligibility criteria are provided in the supplementary methods. The study was conducted according to ethical guidelines at our institution and the Helsinki Declaration and was approved by the ethics committee II of the University of Heidelberg (reference number 2018-653N-MA).

### Treatment procedures

Extracorporeal photopheresis was applied on two consecutive days every two weeks (equals one cycle). Patient 1 intermittently received ECP cycles every three weeks due to the good clinical response. DMF was administered as described before [24]. Topical steroids were allowed as concomitant treatment, but neither systemic steroids nor any other systemic CTCL therapies or topical chemotherapy.

### Clinical assessments

In the four SS patients receiving the DMF/ECP combination therapy, safety was monitored during the regular patient visits. Adverse events were assessed in accordance with the National Cancer Institute Common Terminology Criteria for AEs (CTCAEs) version 5.0. Responses were assessed based on the consensus global response criteria for CTCL [32]. For quantitative assessment of skin disease burden, the modified severity weighted assessment tool (mSWAT) was used in all patients. Blood involvement was measured quantitatively with flow cytometry. Due to the observational nature of the study, no regular study-specific CT scans were performed because of the exposure to radiation. The overall global response was calculated as proposed by Olsen et al. [32], and the TTNT was defined as time to next systemic CTCL treatment according to Campbell et al. [15].

### Statistical analysis

Data were statistically evaluated with GraphPad Prism 8.0. Data are presented as mean ± standard deviation and statistical significance is indicated using *p*-values (*p < 0.05; **p < 0.01; ***p < 0.001; ****p < 0.0001). A two-tailed Student’s *t* test was performed to compare two groups. One-way analysis of variance (ANOVA) Tukey’s multiple comparison test, and two-way ANOVA Tukey’s multiple comparison test were used to compare between multiple conditions, timepoints and data sets.

All methodologies of experiments only presented in the supplementary material is provided there.

## Results

### Combination treatment with DMF and 8-MOP/UVA induces increased cell death in SS patient-derived CD4^+^ T cells and CTCL cell lines in vitro

**Fig. 1:**
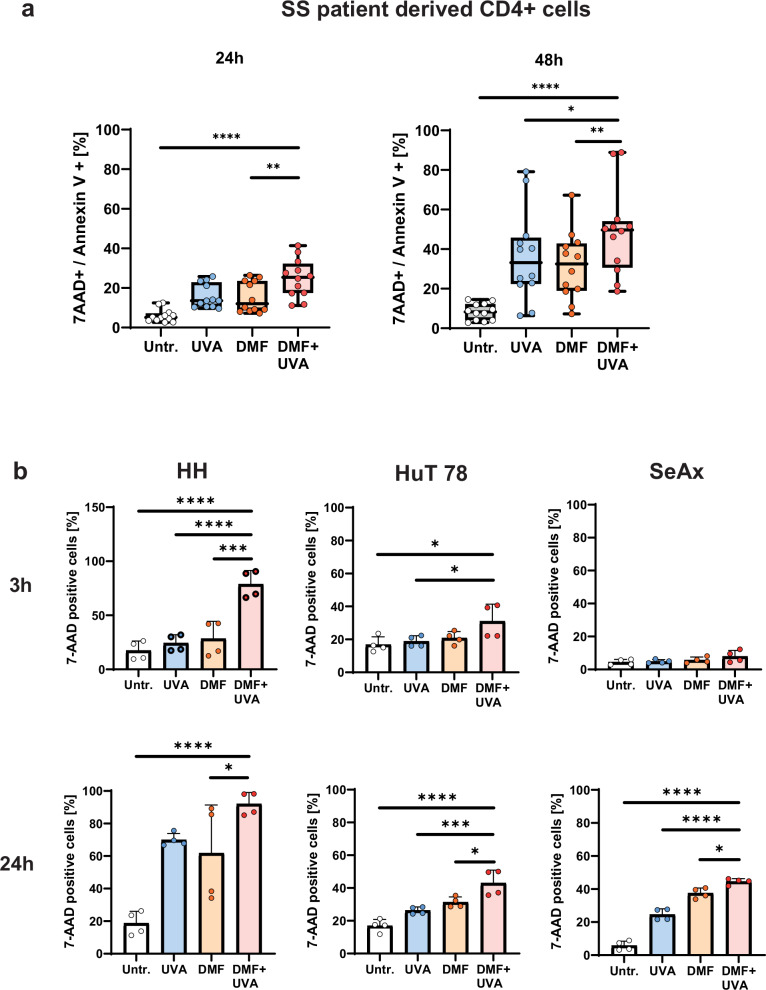
DMF-8-MOP/UVA combination treatment induces increased cell death in SS patient-derived CD4^+^ T cells and CTCL cell lines in vitro. **a** CD4^+^ T cells were isolated from the peripheral blood of SS patients (*n* = 6, the patients’ cells were measured at *n* = 2 different timepoints resulting in overall 12 data points) and treated with DMF- and 8-MOP/UVA-monotreatment, and the DMF-8-MOP/UVA-combination treatment, and compared to untreated controls. Cell death was measured by 7AAD/Annexin V staining with FACS after 24 h and 48 h. **b** HH, HuT 78 and SeAx cells were treated with DMF- and 8-MOP/UVA-monotreatment, and the DMF-8-MOP/UVA-combination treatment, and compared to untreated controls. Cell death was measured by 7AAD staining with FACS after 3 h and 24 h (*n* = 4). The level of significance is indicated by asterisks (**** ≤0.0001; *** ≤0.001; ** ≤0.01; * ≤0.05). Error bars show the standard deviation.

Initially, the DMF and 8-MOP/UVA combination-induced CTCL cell death behavior was investigated in CTCL-patient derived SS cells in order to bring the proof of principle of a synergistic effect of the DMF/ECP combination therapy. CTCL-patient derived CD4^+^ T cells were treated ex vivo with DMF and exposed to UVA irradiation in the presence of 8-MOP to reflect ECP therapy, and cell death was measured. The exact conditions and concentrations are provided in the Supplementary Table 1. The combination treatment of DMF and 8-MOP/UVA induced a significantly higher cell death response compared to the monotreatments in the CD4^+^ T cells isolated from six SS patients ex vivo (Fig. 1a) confirming the combinatory therapeutic effect of DMF and ECP.

To further underline these findings and to create a tool for further mechanistic investigations, the CTCL cell lines HH, HuT 78, and SeAx were used as model systems and treated in vitro with DMF and 8-MOP/UVA as described above. The individual dosages for the detection of the synergistic cell death effect were determined for each cell line (supplementary Fig. 1). The early cell death response was measured after 3 h, and the late cell death response after 24 h (Fig. 1b). After 3 h, a significantly higher cell death response was detected in HH upon the combination treatment with DMF and ECP compared to the monotreatments and the untreated control. Also, in HuT 78 cells, a higher cell death response upon combination treatment was observed compared to 8-MOP/UVA monotreatment and the untreated control, although the effect was not as prominent as in HH cells. Both in HH and HuT 78 cells, corresponding results were measured after 24 h of in vitro treatment. In SeAx cells, a significantly increased cell death upon the combination treatment was observed after 24 h reflecting the lower NF-κB activity compared to HH and HuT 78 cells.

### Transcriptomic analysis of HH cells upon in vitro DMF and 8-MOP/UVA mono- and combination treatment

The in vitro combination treatment with DMF and 8-MOP/UVA induced the highest cell death response in HH cells compared to the other CTCL cell lines. In the 7-AAD stainings, a cell death rate of 70–80% was detected in HH cells upon combination treatment with DMF and 8-MOP/UVA for 3 h (Supplementary Fig. 2a). Consequently, a transcriptomics analysis was conducted and the differentially expressed genes of HH cells upon mono- and combination treatment with DMF and 8-MOP/UVA were determined (Supplementary Fig. 2b). Solute Carrier Family 9 Member A1 (*Slc9a1*) and Activating Transcription Factor 3 (*Atf3*) were significantly increased in expression upon the combination treatment. Compared to DMF monotreatment, the combination treatment exhibited significantly higher expression of the Regulator of G-protein signaling 2 (*Rgs2*) gene. Furthermore, upon 8-MOP/UVA monotreatment, Sphingosine-1-phosphate receptor 1 (*S1pr1*) was significantly lower expressed compared to the combination treatment. Additionally, the mRNA expression of the identified genes was measured in HH, HuT 78, and SeAx cells (Supplementary Fig. 2c). The qPCR results confirmed that *Atf3*, *Rgs2* and *Slc9a1* mRNA were upregulated upon combination treatment with DMF and 8-MOP/UVA in all three CTCL cell lines. *S1pr1* mRNA was significantly downregulated in HuT 78 and SeAx cells, however, in HH cells a trend towards decreased *S1pr1* mRNA expression was detected, although not reaching statistical significance. These findings illustrate that the combination treatment shapes the expression of genes involved in apoptosis, immune cell infiltration and migration, angiogenesis, and the MAPK signaling pathway [33–38].

### NF-κB expression is decreased by the combination treatment with DMF and 8-MOP/UVA compared to DMF and 8-MOP/UVA monotreatment

**Fig. 2:**
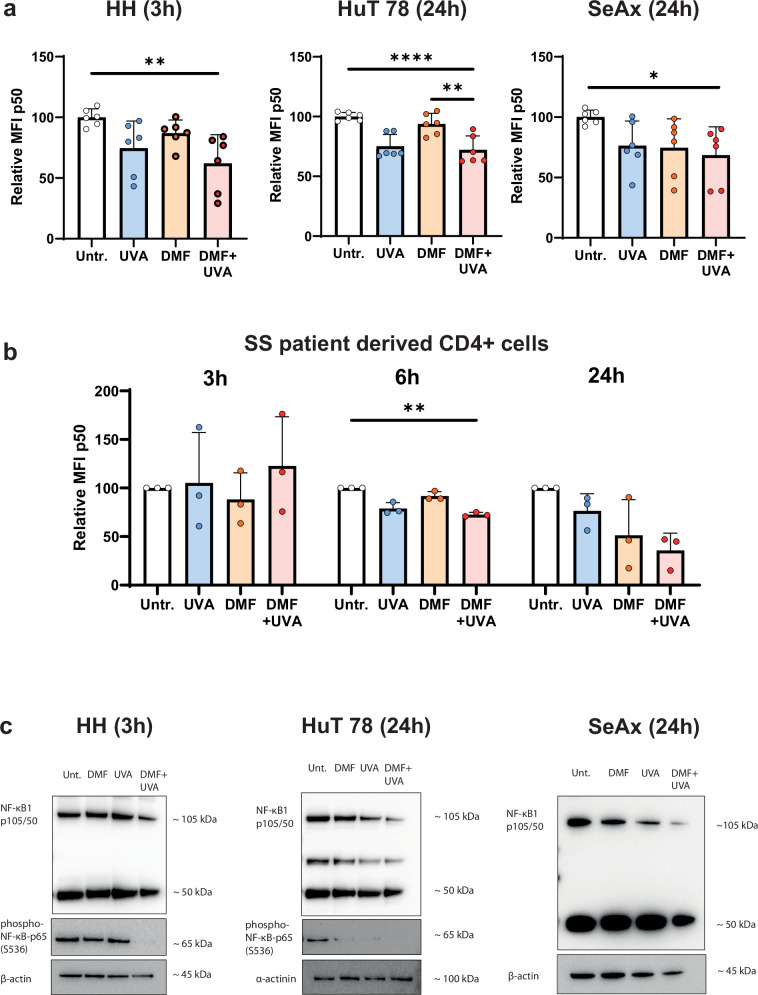
DMF-8-MOP/UVA combination treatment significantly decreases NF-κB expression in CTCL cell lines and SS patient cells compared to monotreatment. HH, HuT 78 and SeAx cells as well as patient-derived CD4^+^ T cells were treated with DMF- and 8-MOP/UVA-monotreatment, and the DMF-8-MOP/UVA-combination treatment, and compared to untreated controls. **a** A p105/p50 staining was performed and the relative MFI was measured by FACS after 3 h for HH cells, and after 24 h for HuT 78 and SeAx cells. **b** The MFI of p105/p50 was measured in patient CD4^+^ T cells after 3 h, 6 h and 24 h. Error bars show the standard deviation (*n* = 6 for cell lines and *n* = 3 patients). The level of significance is indicated by asterisks (**** ≤0.0001; *** ≤0.001; ** ≤0.01; * ≤0.05). **c** NF-κB1 p105/50 and phospho-NF-κB-p65 (S536) were detected in the cell lines by immunoblot (representative immunoblot, *n* = 3).

DMF is a potent NF-κB inhibitor both in activated and malignant cells [26, 39–42]. NF-κB acts as a pro-survival factor and contributes to cell death resistance in many malignant entities including CTCL [43–46]. Therefore, the effect of the combination treatment with DMF and ECP on NF-κB activity was investigated in CTCL cell lines and in CD4^+^ T cells collected from three SS patients. The expression of the canonical NF-κB heterodimers responsible for transactivation NF-κB1 (p105/p50) and phosphorylated p65 (p-p65) were measured in the CTCL cell lines by flow cytometry (Fig. 2a and Supplementary Fig. 3). Subsequently, immunoblot analyses confirmed that the combination treatment of DMF and 8-MOP/UVA significantly reduced p105/50 in all CTCL cell lines. Additionally, HH and HuT 78 cells showed a tendency towards decreased p-p65 expression upon combination treatment. In HH cells, p-p65 expression was completely absent upon combination treatment with DMF and 8-MOP/UVA, while the levels of p105/50 remained unchanged. HuT 78 cells showed a significant decrease in both p105/p50 and p-p65 expression, and SeAx cells showed a decrease in p105/p50, while p-p65 was not detectable (Fig. 2c and Supplementary Fig. 4). These findings demonstrate that a combination treatment with DMF and ECP significantly decreases NF-κB components and suppresses NF-κB activity compared to monotreatment.

In the SS patients, p105/50 significantly decreased after 6 h and after 24 h by an average of 65% following combination treatment (Fig. 2b). Staining for p-p65 also showed a significant decrease 24 h after the combination treatment. In general, the combination treatment resulted in the greatest reduction in both p-p65 and p105/p50 levels compared to DMF and 8-MOP/UVA monotreatment.

### The combination treatment with DMF and 8-MOP/UVA leads to ROS production via decreased Thioredoxin reductase activity and diminished free glutathione levels

**Fig. 3:**
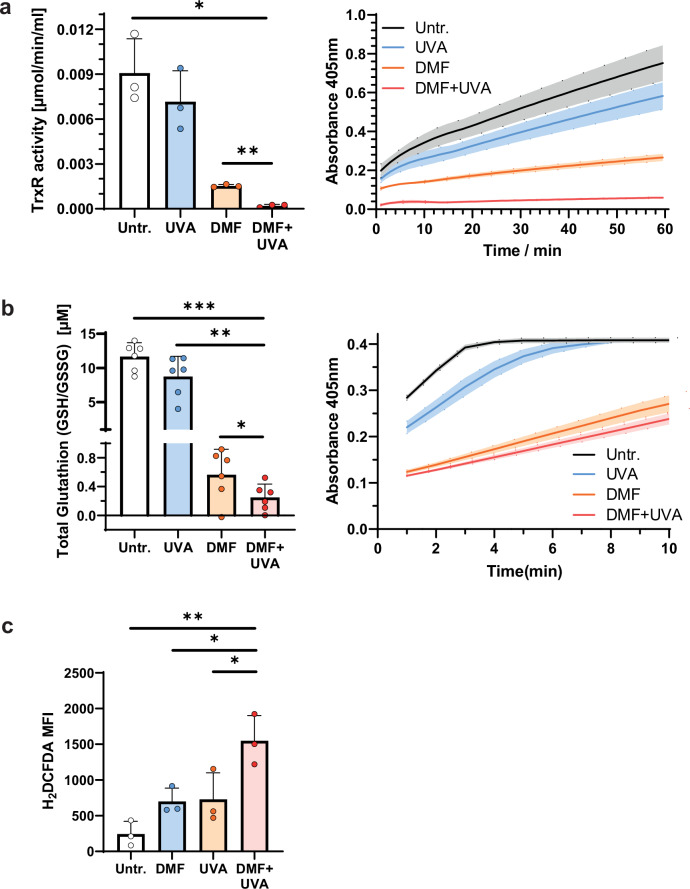
DMF-8-MOP/UVA combination treatment induces ROS production via decreased Thioredoxin reductase activity and diminished free glutathione levels. HH cells were treated with DMF- and 8-MOP/UVA-monotreatment, and the DMF-8-MOP/UVA-combination treatment, and compared to untreated controls. **a** Thioredoxin reductase activity was calculated *via* the increase in absorbance at a wavelength of 405 nm over the course of 60 min according to manufacturer’s instructions (*n* = 3). **b** The concentration of total glutathione (GSSG/GSH) was calculated *via* the increase in absorbance at 405 nm wavelength over the course of 10 min according to manufacturer’s protocol (*n* = 6). **c** The MFI of intracellular H_2_DCFDA was measured by FACS as a surrogate parameter for intracellular reactive oxygen species (ROS) levels (*n* = 3). The level of significance is indicated by asterisks (**** ≤0.0001; *** ≤0.001; ** ≤0.01; * ≤0.05). Error bars show the standard deviation.

In CTCL cells, DMF inhibits NF-κB in part by covalently modifying a thiol group of the redox regulator Trx-1 [14]. Based on these findings, we further investigated the effect of the combination treatment with DMF and 8-MOP/UVA on Trx Reductase (TrxR) activity. Upon combination treatment with DMF and 8-MOP/UVA, TrxR activity was significantly decreased compared to monotreatment and untreated controls in HH cells (Fig. 3a). Similar to the Trx system, the glutathione system provides reduction equivalents and assists in maintaining the redox balance and contributes to increased cell survival in cancer. As observed for the TrxR activity, the levels of glutathione were significantly reduced upon the combination treatment with DMF and 8-MOP/UVA (Fig. 3b).

Subsequently, intracellular reactive oxygen species (ROS) were determined in HH cells by H_2_DCFDA dye (Fig. 3c). The combination treatment led to a significant increase in ROS levels compared to the monotreatments and the untreated controls. Monotreatment with DMF or 8-MOP/UVA did not lead to significantly increased ROS production compared to the untreated controls.

### DMF and 8-MOP/UVA combination treatment induces cell death in CTCL cells *via* different mechanisms and pathways and shapes a pro-inflammatory TME

**Fig. 4:**
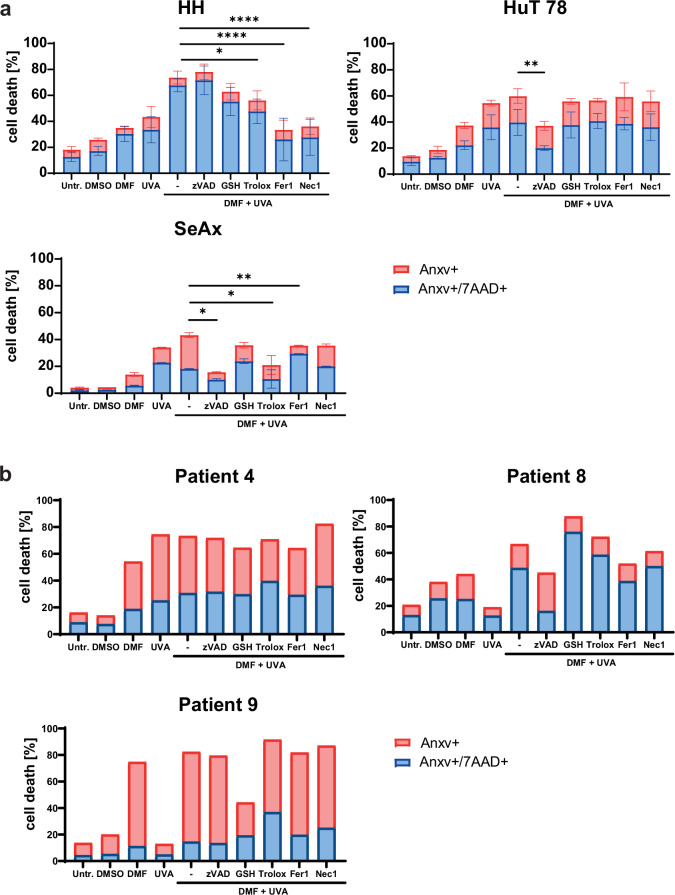
DMF-8-MOP/UVA-combination treatment induces cell death in CTCL cell lines and SS patient cells *via* different mechanisms and pathways. **a** HH, HuT 78 and SeAx cells as well as (**b**) CD4^+^ T cells from three patients were treated with DMF- and 8-MOP/UVA-monotreatment, and the DMF-8-MOP/UVA-combination treatment. Additionally, the combination treatment was performed together with the cell death inhibitors zVAD (50 µM), Ferrostatin 1 (1 µM), Necrostatin 1 (50 µM), and the anti-oxidants Trolox (1 mM) and glutathione (GSH; 0.5 mM), and compared to untreated controls. Cell death was measured by Annexin V/7-AAD staining (*n* = 4 for cell lines). The level of significance is indicated by asterisks (**** ≤0.0001; *** ≤0.001; ** ≤0.01; * ≤0.05). Error bars show the standard deviation.

Consequently, the effects of the combination treatment with DMF and ECP on different cell death mechanisms like apoptosis, necroptosis, and ferroptosis were investigated by in vitro application of specific cell death inhibitors and antioxidants in CTCL cell lines and in CD4^+^ T cells collected from three SS patients. The cotreatment factors used were the pancaspase inhibitor z-VAD-FMK (zVAD; 50 µM), the antioxidants glutathione (GSH; 0.5 mM) and Trolox (1 mM), as well as the ferroptosis inhibitor Ferrostatin-1 (Fer-1; 1 µM), and the Receptor-interacting serine/threonine-protein kinase 1 (RIPK-1) inhibitor Necrostatin-1 (Nec1; 50 µM). HH cells were treated for 3 h, whereas HuT 78, SeAx and SS patient cells were treated for 24 h with DMF and 8-MOP/UVA as mono- and combination treatments together with the aforementioned inhibitors and anti-oxidants (Fig. 4a).

Each CTCL cell line displayed a distinct pattern of susceptibility to cell death that is in line with their differential mutational and aberrant signaling profiles. In HH cells, caspase-independent cell death was identified. Trolox significantly reduced the cell death induced by the DMF-8-MOP/UVA combination which points towards a causative role of oxidative stress upon combination treatment with DMF and 8-MOP/UVA. Additionally, the cell death induction by the combination treatment was rescued by Fer-1 and Nec-1, indicating cell death induction via necroptosis and ferroptosis.

In HuT 78 cells, cell death induction by the combination treatment with DMF and 8-MOP/UVA was significantly reduced by application of the pan-caspase inhibitor zVAD which points towards caspase-dependent cell death. In SeAx cells, cell death upon the combination treatment with DMF and 8-MOP/UVA was rescued by the application of zVAD and Trolox. This indicates that in SeAx cells, the combination treatment with DMF and 8-MOP/UVA induces cell death both through caspase-dependent pathways and oxidative stress.

The same observation was made for three SS patients (Fig. 4b). While CD4^+^ T cells from each patient responded strongly to the combination treatment, different mechanisms of cell death were observed to be involved. CD4^+^ T cells from patient 4 were more sensitive to 8-MOP/UVA treatment compared to DMF, in contrast to the other patients. Surprisingly, cell death could not be reversed by any inhibitors or antioxidants. Cells from patient 8 showed reduced cell death after zVAD and Fer-1 application, indicating caspase-dependent cell death and ferroptosis. Interestingly, the antioxidant Trolox and GSH rather promoted cell death. In patient 9, cell death could be prevented by GSH treatment, revealing oxidative stress as one mechanism.

**Fig. 5:**
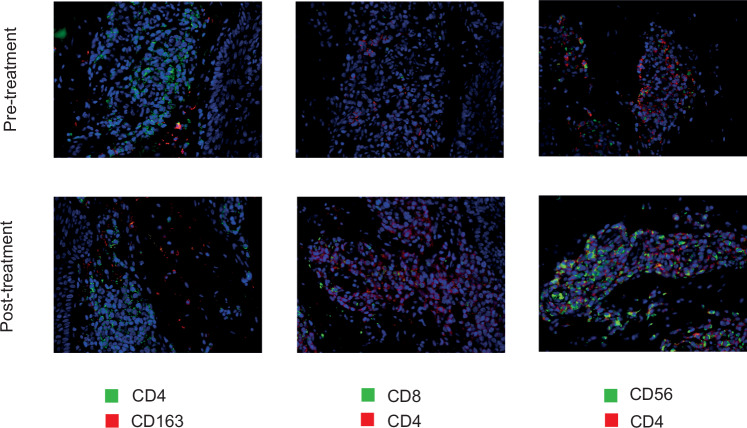
Immunofluorescent stainings of patient 1, patient 2, and patient 3 before and under the DMF/ECP combination treatment. Representative pictures of immunofluorescent stainings of patient 1, patient 2, and patient 3 before and under the DMF/ECP combination treatment stained for CD4, CD8, CD163, CD56, and with DAPI, the latter to counterstain nuclei.

Immunofluorescent stainings for CD4, CD8, CD163, and CD56 were prepared from lesional skin from patient 1, patient 2, and patient 3 before and under DMF/ECP combination therapy. The immunofluorescent stainings revealed an increase in CD8^+^ T cells, macrophages and NK cells under DMF/ECP-combination therapy and hinting a shift towards a more pro-inflammatory tumor microenvironment under DMF/ECP-combination therapy (Fig. 5).

These findings illustrate how the DMF/ECP-combination treatment influences different molecular mechanisms, shapes the TME and induces different cell death pathways. This explains why all patients responded to the combination treatment and underlines its high efficacy in CTCL cells despite their high mutational heterogeneity and molecular aberrations.

### DMF and ECP combination therapy shows excellent responses in skin and blood and good tolerability in SS patients

**Fig. 6:**
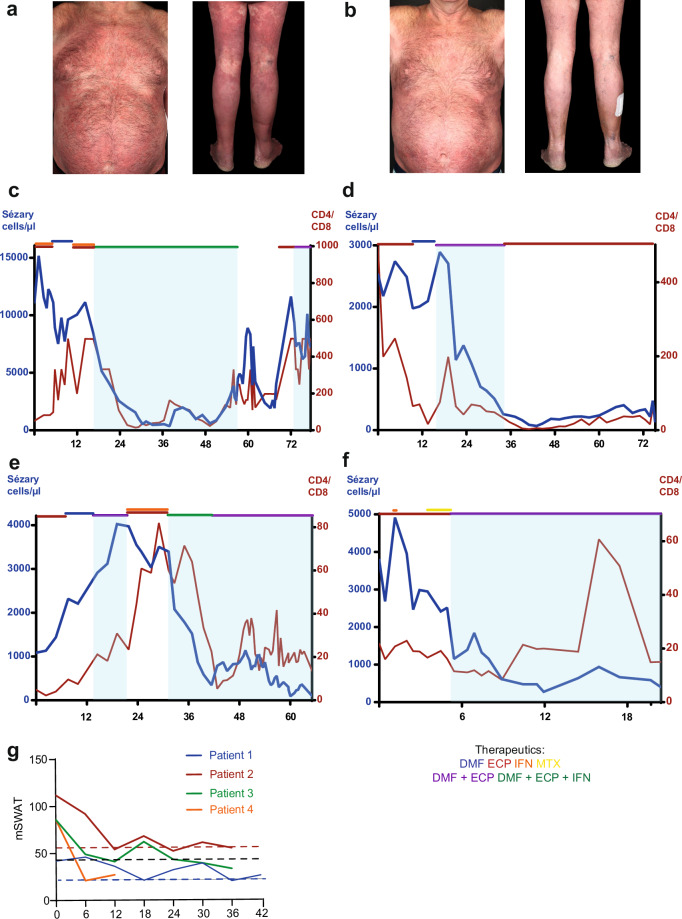
DMF and ECP combination therapy shows excellent clinical responses in skin and blood in SS patients. Clinical photographs from patient 2 at week 0 (**a**) and week 52 (**b**) of the combination treatment of dimethyl fumarate (DMF) and extracorporeal photopheresis (ECP). Course of the Sézary cells (CD3^+^CD4^+^CD7- cell population; in blue) and the CD4/CD8 ratio (red) from patient 1 (**c**), patient 2 (**d**), patient 3 (**e**) and patient 4 (**f**) over time (in months). The patients’ treatments since the introduction of ECP in the therapeutic regimen are marked with colored lines (DMF = blue, ECP = red, IFN = orange, MTX = yellow, combination therapy with DMF and ECP = purple, combination therapy with DMF, ECP, and IFN = green). The period of combination therapy with DMF and ECP is shaded in light blue. **g** mSWAT scores during the combination therapy of DMF and ECP of patient 1 (blue), patient 2 (red), patient 3 (green), and patient 4 (orange) over time (in months).

Following the promising preclinical results, four SS patients were treated with this novel combination therapy of DMF and ECP. The overall response rate in the skin and blood compartment was 100%. In the skin compartment, all four patients had a stable partial response, while in the peripheral blood, three of four patients had a complete response, and one patient had a very good partial response (Sézary cells (SC) 276/µl (B1)) (Fig. 6).

Of note, all of the patients experienced an added clinical benefit from the DMF/ECP-combination therapy compared to the monotherapy. This observation also applies for the DMF/IFN/ECP-combination therapy, since patient 1 and patient 3 were pre-treated with IFN/ECP-combination therapy before without comparable clinical response, but rather resulting in disease progression. Patient 1 only reached complete remission in the blood after addition of DMF to the IFN/ECP-combination therapy, and in patient 3, the DMF/IFN/ECP-combination therapy was terminated after ten months, and the patient received a maintenance therapy with DMF/ECP for additional 24 months.

The TTNT was highly individual due to the heterogeneous disease courses. For patient 1, the TTNT for the initial combination therapy with DMF, ECP, and interferon was 39 months, and for the rechallenge with DMF and ECP, 6 months. The TTNT for patient 2 was 19 months for the DMF/ECP combination therapy, and 57 months for the maintenance therapy with ECP monotherapy. Patient 3 had a TTNT of 9 months for the initial DMF/ECP combination therapy, and 24 months for the rechallenge with DMF and ECP in the later disease course. Patient 4 is still under the combination therapy with ECP and DMF for 16 months (Table 1). A detailed therapy course since the first introduction of ECP in the therapeutic algorithm is provided in supplementary Table 3.

**Table 1:** Time to next treatment of the DMF and ECP combination therapy in SS patients.

Patient	Time to next treatment
Patient 1	39 months for the initial combination therapy with DMF, ECP and IFN, and 6 months for the rechallenge with DMF and ECP
Patient 2	19 months for the combination therapy with DMF and ECP, 57 months for ECP maintenance therapy
Patient 3	9 months for the initial therapy and 24 months for the rechallenge with DMF and ECP
Patient 4	preliminary 16 months (TTNT not yet reached)

In summary, all patients had at least once a TTNT of more than 16 months which exceeds the median TTNT of ECP-based combination therapies, that are not administered as first line treatment, by 9 months [15]. Thus, our data present the DMF/ECP combination therapy as an initially and long-lasting highly effective therapy.

The adverse events (AE) were assessed during the regular patient visits in our clinic. All four patients experienced AEs. Most AEs concerned laboratory findings like peripheral bood count changes or transaminase elevations. Two patients had an AE ≥ CTCAE grade 3, in both cases lymphocytopenia, a common side effect of DMF treatment. In one patient, the DMF/ECP combination treatment was switched to an ECP maintenance therapy due to lymphocytopenia CTCAE grade 3 and the good clinical response in the skin and blood compartment (PR in the skin, CR in the blood). All other AE were rather mild and included mainly typical DMF side effects and did not lead to drug interruption or dosage reduction. All AEs are provided in detail in Table 2. The exact individual therapy regimens and courses are depicted in detail in Supplementary Table 4.

**Table 2:** Adverse events of the DMF and ECP combination therapy in SS patients.

Adverse event	CTCAE grade	Patients [%]	Patient count
Bullous dermatitis	2	25	1/4
Urticaria	2	25	1/4
Hyperkalemia	1	50	2/4
Blood lactate dehydrogenase increased	1	100	4/4
Alanine aminotransferase increased	1	50	2/4
Aspartate aminotransferase increased	1	25	1/4
Gamma-glutamyltransferase increased	1	50	2/4
Anemia	1	75	3/4
	2	25	1/4
Platelet count decreased	2	25	1/4
Neutrophil count decreased	1	25	1/4
Lymphocyte count decreased	1	25	1/4
	2	25	1/4
	3	50	2/4
Proteinuria	1	75	3/4
Hematuria	1	75	3/4
Diarrhea	1	25	1/4
Flushing	1	25	1/4

## Discussion

CTCL remains a therapeutic challenge due to high relapse rates upon initially highly effective therapies. Combinatory therapeutic regimens are shown to be beneficial and are increasingly applied to induce long-term clinical responses [14, 15]. However, the underlying molecular mechanisms especially of suspected synergistic effects between the applied therapeutics have not yet been identified. Our study elucidates the molecular mechanisms behind the synergistic therapeutic effects of the DMF/ECP combination therapy.

Our preclinical experiments revealed that the combination of DMF and 8-MOP/UVA irradiation significantly induced cell death in purified cells from SS patients, as well as in three different CTCL cell lines in vitro. The constitutive activity of NF-κB can hinder cell death signaling in CTCL cells by causing a lack of PP4R1, which disrupts the controlled NF-κB signaling by interfering with its assembly with PP4c, but also other more indirect ways [28]. Our findings indicate that the combination of DMF and 8-MOP/UVA can synergistically target NF-κB more effectively than either treatment alone.

In CTCL cells, DMF reduces the activity of Trx1 and consequently decreases NF-κB signaling [27]. In our study, DMF and 8-MOP/UVA combination treatment is more effective in reducing both TrxR activity and Glutathione levels than DMF monotreatment in HH cells. The findings indicate that the decrease in TrxR activity and Glutathione levels leads to the reduction of free thiols in cysteine residues in various proteins and peptides, including NF-κB components, and also decreases their transcriptional and redox activity. The inhibition of TrxR can disrupt the antioxidant regulation of neoplastic cells and lead to a significant increase in ROS generation in CTCL cells when treated with DMF and 8-MOP/UVA. Elevated intracellular levels of ROS can lead to oxidative stress and to the degradation of cellular components such as DNA, lipids, and proteins, ultimately resulting in cell death. TrxR inhibits apoptosis which makes TrxR inhibition a promising therapeutic strategy in oncology [47]. Additionally, depletion of Glutathione renders neoplastic cells more susceptible to ROS-based therapies like chemo-, and phototherapy [48].

Our study identified various mechanisms contributing to cell death induction, including ferroptosis, RIPK1-dependent necroptosis, and caspase-dependent apoptosis in different CTCL cell lines. In CD4^+^ T cells collected from SS patients, caspase-dependent cell death, ferroptosis and oxidative stress seemed to be inducible by DMF/8-MOP-UVA treatment. The inhibition of constitutively activated NF-κB leads to increased intracellular iron levels, which can induce a significant spike in the generation of ROS in CTCL. The combination treatment of DMF and 8-MOP/UVA induces ferroptosis and necroptosis in HH cells indicating that oxidative stress-induced cell death mechanisms were triggered by extensive production of ROS. Ferroptosis is a crucial cell death response triggered by various therapies [49]. For DLBCL, Schmitt et al. were able to show that DMF induces ferroptosis and impairs NF-κB/STAT3 signaling [50]. In contrast to HH cells, HuT 78 and SeAx cells showed significantly decreased cell death upon the combination treatment with DMF and 8-MOP/UVA when co-treated with zVAD, indicating caspase-dependent cell death in these cell lines (Fig. 5). In previous studies, hyperactive RAS-RAF signaling was detected in HuT 78 cells, and MEK inhibitors were able to induce apoptosis in these cells, which makes MEK inhibition a potential new therapeutic target for future studies [29]. Overall, the combination of DMF and 8-MOP/UVA induces various cell death pathways, making it challenging for cancer cells to evade.

Additionally, a transcriptome analysis of HH cells treated with the 8-MOP/UVA, DMF or the combination treatment revealed several differentially expressed genes related to immune cell infiltration, apoptosis, and angiogenesis. All the aberrations in the expression of these genes collectively suggest a strong effect of the ECP/DMF combination treatment on immunological properties and microenvironmental stimuli of CTCL cells.

In the clinical setting, combinatory therapeutic regimens are increasingly applied to induce long-term clinical responses [14, 15]. CTCL is characterized by a very short median TTNT of 5.4 months [12, 15], while first-line ECP-based combination therapies were reported to have a median TTNT of 9.8 months, which decreased to a TTNT of 6.7 months for ECP-based combination therapies across all treatment lines [15]. Our study introduces the combination therapy of DMF and ECP as a very effective treatment with an excellent ORR of 100%, and a good tolerability. The TTNT of the DMF/ECP-combination was more than twice as high than the median TTNT for ECP-based combination therapies [15]. Thus, our study is the first one to investigate DMF-based combination therapies in CTCL patients.

So far, only limited data on the efficacy and safety of combinatory therapeutic regimens exist. The combination of ECP with the anti-CCR4 antibody Mogamulizumab achieved an ORR of 73% in the skin compartment and an ORR of 64% in the blood compartment in the study of Ninosu et al. with 11 patients [16]. The combination treatment with ECP and IFN is another combinatory regimen in CTCL that has long been in clinical practice. In the study of Dippel et al. from 1997, ten patients were treated with ECP monotreatment, and nine patients were treated with a combination of ECP and IFN. The patients receiving the combination treatment with ECP and IFN had an ORR of 67% compared to an ORR of 20% for ECP monotherapy [14].

In our study on the combination treatment with ECP and DMF, we report an ORR of 100% both in the skin and the blood compartment, and a TTNT in all patients at least once for more than 16 months and up to 56 months. Thus, both ORR and TTNT are higher compared to other combinatory regimens. Additionally, all of the patients experienced an added clinical benefit from the DMF/ECP-combination therapy compared to the monotherapy. Comparing safety to other combinatory regiments is difficult and subject to bias since a systematic AE reporting was not conducted quantitatively in most case series. Additionally, attributing single AE individually to the respective combination partners or the combination itself is also biased and thus questionable.

As our pilot study only includes four patients it bears limitations in extrapolating its results to the whole CTCL patient population. The current number of patients is limited and the samples were not collected in a standardized way within a clinical trial. Therefore, the present study is a translational pilot to be followed by further prospective clinical evaluation with a translational research part that gathers more patient samples in a standardized manner that is being planned at the moment.

In summary, we present in our study the DMF/ECP combination therapy as an effective and well-tolerable treatment in four CTCL patients. Furthermore, we demonstrate that this combination synergistically induces cell death in CTCL. Translational results reveal that DMF and ECP induce a wide variety of cell death mechanisms and can therefore be broadly effective against CTCL cells despite their high mutational heterogeneity that complicates other therapies. Additionally, these findings stress the importance of a combinatory therapeutic approach for CTCL patients. These advances hold promise for improving treatment outcomes and prognosis of CTCL patients, offering new avenues for more effective and personalized therapies.

## Supplementary information

Supplements

## Data Availability

The raw data of the transcriptomics analysis in this study have been deposited in the NCBI Sequence Read Archive (SRA) under the accession number PRJNA1117212 and made publicly available. Additional supporting data from this study are available in the online version of this article (Supporting information) and from the corresponding author upon request.

## References

[1] Alaggio R, Amador C, Anagnostopoulos I, Attygalle AD, Araujo IBO, Berti E, et al. The 5th edition of the World Health Organization Classification of Haematolymphoid Tumours: Lymphoid Neoplasms. Leukemia. 2022;36:1720–48. 35732829 10.1038/s41375-022-01620-2 PMC9214472

[2] Willemze R, Cerroni L, Kempf W, Berti E, Facchetti F, Swerdlow SH, et al. The 2018 update of the WHO-EORTC classification for primary cutaneous lymphomas. Blood. 2019;133:1703–14. 30635287 10.1182/blood-2018-11-881268 PMC6473500

[3] Latzka J, Assaf C, Bagot M, Cozzio A, Dummer R, Guenova E, et al. EORTC consensus recommendations for the treatment of mycosis fungoides/Sezary syndrome - Update 2023. Eur J Cancer. 2023;195:113343. 37890355 10.1016/j.ejca.2023.113343

[4] Willemze R, Hodak E, Zinzani PL, Specht L, Ladetto M, Committee EG. Primary cutaneous lymphomas: ESMO Clinical Practice Guidelines for diagnosis, treatment and follow-up. Ann Oncol. 2018;29:iv30–iv40. 29878045 10.1093/annonc/mdy133

[5] Knobler R, Arenberger P, Arun A, Assaf C, Bagot M, Berlin G, et al. European dermatology forum: Updated guidelines on the use of extracorporeal photopheresis 2020 - Part 2. J Eur Acad Dermatol Venereol. 2021;35:27–49. 32964529 10.1111/jdv.16889 PMC7821314

[6] Edelson R, Berger C, Gasparro F, Jegasothy B, Heald P, Wintroub B, et al. Treatment of cutaneous T-cell lymphoma by extracorporeal photochemotherapy. Preliminary results. N Engl J Med. 1987;316:297–303. 3543674 10.1056/NEJM198702053160603

[7] Bladon J, Taylor P. Extracorporeal photopheresis reduces the number of mononuclear cells that produce pro-inflammatory cytokines, when tested ex-vivo. J Clin Apher. 2002;17:177–82. 12494410 10.1002/jca.10039

[8] Bladon J, Taylor PC. Lymphocytes treated by extracorporeal photopheresis can down-regulate cytokine production in untreated monocytes. Photodermatol Photoimmunol Photomed. 2005;21:293–302. 16313240 10.1111/j.1600-0781.2005.00192.x

[9] Bladon J, Taylor PC. Extracorporeal photopheresis: a focus on apoptosis and cytokines. J Dermatological Sci. 2006;43:85–94. 10.1016/j.jdermsci.2006.05.004 16797926

[10] Tsai YC, Schlaepfer T, Ignatova D, Chang YT, Valaperti A, Amarov B, et al. Boost of innate immunity cytokines as biomarkers of response to extracorporeal photopheresis in leukaemic cutaneous T-cell lymphoma patients. Br J Dermatol. 2023;189:603–611. 10.1093/bjd/ljad220 PMC13077219 37409661

[11] Plumas J, Manches O, Chaperot L. Mechanisms of action of extracorporeal photochemotherapy in the control of GVHD: involvement of dendritic cells. Leukemia. 2003;17:2061–2. 12949576 10.1038/sj.leu.2403114

[12] Hughes CF, Khot A, McCormack C, Lade S, Westerman DA, Twigger R, et al. Lack of durable disease control with chemotherapy for mycosis fungoides and Sezary syndrome: a comparative study of systemic therapy. Blood. 2015;125:71–81. 25336628 10.1182/blood-2014-07-588236

[13] Nicolay JP, Felcht M, Schledzewski K, Goerdt S, Geraud C. Sezary syndrome: old enigmas, new targets. J Dtsch Dermatol Ges. 2016;14:256–64. 26972187 10.1111/ddg.12900

[14] Dippel E, Schrag H, Goerdt S, Orfanos CE. Extracorporeal photopheresis and interferon-alpha in advanced cutaneous T-cell lymphoma. Lancet. 1997;350:32–3. 9217723 10.1016/s0140-6736(05)66242-3

[15] Campbell BA, Dobos G, Haider Z, Prince HM, Bagot M, Evison F, et al. International Study of SS Shows Superiority of Combination Therapy & Heterogeneity of Treatment Strategies. Blood Adv. 2023;7:6639–47. 10.1182/bloodadvances.2023011041 PMC10628811 37648672

[16] Ninosu N, Melchers S, Kappenstein M, Booken N, Hansen I, Blanchard M, et al. Mogamulizumab Combined with Extracorporeal Photopheresis as a Novel Therapy in Erythrodermic Cutaneous T-cell Lymphoma. Cancers. 2023;16:141. 10.3390/cancers16010141 PMC10778082 38201568

[17] Rubio-Muniz CA, Sanchez-Velazquez A, Arroyo-Andres J, Agud-de Dios M, Tarin-Vicente EJ, Falkenhain-Lopez D, et al. Mogamulizumab combined with extracorporeal photopheresis for the treatment of refractory mycosis fungoides and Sezary syndrome. Report of seven cases. J Eur Acad Dermatol Venereol. 2024;38:e102–e105. 10.1111/jdv.19457 37611255

[18] Gottlieb SL, Wolfe JT, Fox FE, DeNardo BJ, Macey WH, Bromley PG, et al. Treatment of cutaneous T-cell lymphoma with extracorporeal photopheresis monotherapy and in combination with recombinant interferon alfa: a 10-year experience at a single institution. J Am Acad Dermatol. 1996;35:946–57. 8959954 10.1016/s0190-9622(96)90119-x

[19] Klemke CD, Brenner D, Weiss EM, Schmidt M, Leverkus M, Gulow K, et al. Lack of T-cell receptor-induced signaling is crucial for CD95 ligand up-regulation and protects cutaneous T-cell lymphoma cells from activation-induced cell death. Cancer Res. 2009;69:4175–83. 19435902 10.1158/0008-5472.CAN-08-4631

[20] Meech SJ, Edelson R, Walsh P, Norris DA, Duke RC. Reversible resistance to apoptosis in cutaneous T cell lymphoma. Ann N. Y Acad Sci. 2001;941:46–58. 11594582 10.1111/j.1749-6632.2001.tb03710.x

[21] Ni X, Zhang C, Talpur R, Duvic M. Resistance to activation-induced cell death and bystander cytotoxicity via the Fas/Fas ligand pathway are implicated in the pathogenesis of cutaneous T cell lymphomas. J Invest Dermatol. 2005;124:741–50. 15816832 10.1111/j.0022-202X.2005.23657.x

[22] Patil K, Kuttikrishnan S, Khan AQ, Ahmad F, Alam M, Buddenkotte J, et al. Molecular pathogenesis of Cutaneous T cell Lymphoma: Role of chemokines, cytokines, and dysregulated signaling pathways. Semin Cancer Biol. 2022;86:382–99. 34906723 10.1016/j.semcancer.2021.12.003

[23] Wang L, Ni X, Covington KR, Yang BY, Shiu J, Zhang X, et al. Genomic profiling of Sezary syndrome identifies alterations of key T cell signaling and differentiation genes. Nat Genet. 2015;47:1426–34. 26551670 10.1038/ng.3444 PMC4829974

[24] Nicolay JP, Melchers S, Albrecht JD, Assaf C, Dippel E, Stadler R, et al. Dimethyl fumarate treatment in relapsed and refractory cutaneous T cell lymphoma - a multicenter phase II study. Blood. 2023;142:794–805. 10.1182/blood.2022018669 PMC10644069 37217183

[25] Froehlich TC, Muller-Decker K, Braun JD, Albrecht T, Schroeder A, Gulow K, et al. Combined inhibition of Bcl-2 and NFkappaB synergistically induces cell death in cutaneous T-cell lymphoma. Blood. 2019;134:445–55. 31167801 10.1182/blood.2019001545

[26] Nicolay JP, Muller-Decker K, Schroeder A, Brechmann M, Mobs M, Geraud C, et al. Dimethyl fumarate restores apoptosis sensitivity and inhibits tumor growth and metastasis in CTCL by targeting NF-kappaB. Blood. 2016;128:805–15. 27268084 10.1182/blood-2016-01-694117 PMC5026464

[27] Schroeder A, Warnken U, Roth D, Klika KD, Vobis D, Barnert A, et al. Targeting Thioredoxin-1 by dimethyl fumarate induces ripoptosome-mediated cell death. Sci Rep. 2017;7:43168. 28233787 10.1038/srep43168 PMC5324128

[28] Brechmann M, Mock T, Nickles D, Kiessling M, Weit N, Breuer R, et al. A PP4 holoenzyme balances physiological and oncogenic nuclear factor-kappa B signaling in T lymphocytes. Immunity. 2012;37:697–708. 23084358 10.1016/j.immuni.2012.07.014

[29] Kiessling MK, Nicolay JP, Schlor T, Klemke CD, Suss D, Krammer PH, et al. NRAS mutations in cutaneous T cell lymphoma (CTCL) sensitize tumors towards treatment with the multikinase inhibitor Sorafenib. Oncotarget. 2017;8:45687–97. 28537899 10.18632/oncotarget.17669 PMC5542218

[30] Melchers S, Roemer M, Albrecht JD, Assaf C, von Gugelberg C, Guenova E, et al. Evaluation of Sezary cell marker expression and cell death behaviour upon in vitro treatment by flow cytometry in Sezary syndrome patients. Exp Dermatol. 2024;33:e15171. 39219147 10.1111/exd.15171

[31] Kaminski MM, Sauer SW, Klemke CD, Suss D, Okun JG, Krammer PH, et al. Mitochondrial reactive oxygen species control T cell activation by regulating IL-2 and IL-4 expression: mechanism of ciprofloxacin-mediated immunosuppression. J Immunol. 2010;184:4827–41. 20335530 10.4049/jimmunol.0901662

[32] Olsen EA, Whittaker S, Willemze R, Pinter-Brown L, Foss F, Geskin L, et al. Primary cutaneous lymphoma: recommendations for clinical trial design and staging update from the ISCL, USCLC, and EORTC. Blood. 2022;140:419–37. 34758074 10.1182/blood.2021012057 PMC9353153

[33] Glal D, Sudhakar JN, Lu HH, Liu MC, Chiang HY, Liu YC, et al. ATF3 Sustains IL-22-Induced STAT3 Phosphorylation to Maintain Mucosal Immunity Through Inhibiting Phosphatases. Front Immunol. 2018;9:2522. 30455690 10.3389/fimmu.2018.02522 PMC6230592

[34] Jia Y, Liu W, Zhan HE, Yi XP, Liang H, Zheng QL, et al. Roles of hsa-miR-12462 and SLC9A1 in acute myeloid leukemia. J Hematol Oncol. 2020;13:101. 32703317 10.1186/s13045-020-00935-w PMC7376648

[35] Ku HC, Cheng CF. Master Regulator Activating Transcription Factor 3 (ATF3) in Metabolic Homeostasis and Cancer. Front Endocrinol. 2020;11:556. 10.3389/fendo.2020.00556 PMC7457002 32922364

[36] Lin Q, Liu T, Wang X, Hou G, Xiang Z, Zhang W, et al. Long noncoding RNA HITT coordinates with RGS2 to inhibit PD-L1 translation in T cell immunity. J Clin Invest. 2023;133:e162951. 10.1172/JCI162951 PMC10231998 37014700

[37] Zhang W, Fan W, Guo J, Wang X. Osmotic stress activates RIPK3/MLKL-mediated necroptosis by increasing cytosolic pH through a plasma membrane Na(+)/H(+) exchanger. Sci Signal. 2022;15:eabn5881. 35580168 10.1126/scisignal.abn5881

[38] Zhou YT, Chen H, Ai M, Li SS, Li BY, Zhao Y, et al. Type-1 Na(+)/H(+) exchanger is a prognostic factor and associate with immune infiltration in liver hepatocellular carcinoma. Life Sci. 2021;278:119613. 34000263 10.1016/j.lfs.2021.119613

[39] Gerdes S, Shakery K, Mrowietz U. Dimethylfumarate inhibits nuclear binding of nuclear factor kappaB but not of nuclear factor of activated T cells and CCAAT/enhancer binding protein beta in activated human T cells. Br J Dermatol. 2007;156:838–42. 17381463 10.1111/j.1365-2133.2007.07779.x

[40] Gu B, DeAngelis LM. Enhanced cytotoxicity of bioreductive antitumor agents with dimethyl fumarate in human glioblastoma cells. Anticancer Drugs. 2005;16:167–74. 15655414 10.1097/00001813-200502000-00008

[41] Loewe R, Pillinger M, de Martin R, Mrowietz U, Groger M, Holnthoner W, et al. Dimethylfumarate inhibits tumor-necrosis-factor-induced CD62E expression in an NF-kappa B-dependent manner. J Invest Dermatol. 2001;117:1363–8. 11886496 10.1046/j.0022-202x.2001.01576.x

[42] Loewe R, Valero T, Kremling S, Pratscher B, Kunstfeld R, Pehamberger H, et al. Dimethylfumarate impairs melanoma growth and metastasis. Cancer Res. 2006;66:11888–96. 17178886 10.1158/0008-5472.CAN-06-2397

[43] Annunziata CM, Davis RE, Demchenko Y, Bellamy W, Gabrea A, Zhan F, et al. Frequent engagement of the classical and alternative NF-kappaB pathways by diverse genetic abnormalities in multiple myeloma. Cancer Cell. 2007;12:115–30. 17692804 10.1016/j.ccr.2007.07.004 PMC2730509

[44] Davis RE, Brown KD, Siebenlist U, Staudt LM. Constitutive nuclear factor kappaB activity is required for survival of activated B cell-like diffuse large B cell lymphoma cells. J Exp Med. 2001;194:1861–74. 11748286 10.1084/jem.194.12.1861 PMC2193582

[45] Gallardo F, Bertran J, Lopez-Arribillaga E, Gonzalez J, Menendez S, Sanchez I, et al. Novel phosphorylated TAK1 species with functional impact on NF-kappaB and beta-catenin signaling in human Cutaneous T-cell lymphoma. Leukemia. 2018;32:2211–23. 29511289 10.1038/s41375-018-0066-4 PMC6170395

[46] Weston VJ, Austen B, Wei W, Marston E, Alvi A, Lawson S, et al. Apoptotic resistance to ionizing radiation in pediatric B-precursor acute lymphoblastic leukemia frequently involves increased NF-kappaB survival pathway signaling. Blood. 2004;104:1465–73. 15142883 10.1182/blood-2003-11-4039

[47] Bian M, Fan R, Zhao S, Liu W. Targeting the Thioredoxin System as a Strategy for Cancer Therapy. J Med Chem. 2019;62:7309–21. 30963763 10.1021/acs.jmedchem.8b01595

[48] Niu B, Liao K, Zhou Y, Wen T, Quan G, Pan X, et al. Application of glutathione depletion in cancer therapy: Enhanced ROS-based therapy, ferroptosis, and chemotherapy. Biomaterials. 2021;277:121110. 34482088 10.1016/j.biomaterials.2021.121110

[49] Zhou Q, Li T, Qin Q, Huang X, Wang Y. Ferroptosis in lymphoma: Emerging mechanisms and a novel therapeutic approach. Front Genet. 2022;13:1039951. 36406116 10.3389/fgene.2022.1039951 PMC9669386

[50] Schmitt A, Xu W, Bucher P, Grimm M, Konantz M, Horn H, et al. Dimethyl fumarate induces ferroptosis and impairs NF-kappaB/STAT3 signaling in DLBCL. Blood. 2021;138:871–84. 33876201 10.1182/blood.2020009404

