# Supplementary material for: Dimethyl fumarate and extracorporeal photopheresis combination-therapy synergize in inducing specific cell death and long-term remission in cutaneous T cell lymphoma
Source: Leukemia. 2024 Nov 23;39(2):438–50. doi: 10.1038/s41375-024-02479-1 (PMC11794131; doi:10.1038/s41375-024-02479-1)
Supplement: Supplementary file 1 — Supplements [file 41375_2024_2479_MOESM1_ESM.pdf]

Supplementary information

Supplementary methods

Immunofluorescence

Immunofluorescent stainings were performed on 3-µm paraffin sections that were deparaffinized and heat-induced antigen retrieval was performed in HIER EDTA buffer pH 9 buffer (Zytomed, Berlin, Germany) for 20 min at boiling temperature. Then, the specimens were blocked with the MaxBlock Autofluorescence Reducing Reagent Kit (Biozol) according to the manufacturer's instructions, and 5% donkey serum (Dianova) in PBS for 30 min at room temperature. The sections were incubated with the primary antibodies presented in supplemental table 4 at 4°C overnight. Then the appropriate fluorophore-conjugated secondary antibodies (Cy3- AffiniPure goat anti-mouse IgG, and Alexa Fluor® 488-AffiniPure donkey anti-rabbit IgG; supplementary table 4), and 4′,6-diamidino-2-phenylindole (DAPI) (Invitrogen, Carlsbad, California, USA) were applied for 30 min at room temperature and the slides were mounted with Fluorescent Mounting Medium (Agilent, Santa Clara, California, USA). Sections incubated without primary antibody were included as negative controls. Immunofluorescent stainings were analyzed by a Keyence BZ-X810 microscope (Keyence, Oska, Japan) with the Keyence BZ-X800 Analyzer software (Version 1.1.2.4; Keyence, Oska, Japan).

Total RNA Sequencing

HH cells were treated with DMF, 8-MOP/UVA and the combination treatment of DMF and 8-MOP/UVA as described in the materials and methods section. RNA was isolated with the RNeasy Mini Kit (Qiagen) according to the manufacturer's instructions. Total RNA Sequencing was performed at BGI (Hongkong, China).

Eligibility criteria

Inclusion criteria

- Histopathologically confirmed Sézary syndrome (CTCL stage IV according to EORTC-ISCL consensus classification) with refractory or recurrent disease
- Pretreatment with at least one systemic CTCL therapy
- Age ≥ 18 years
- Ability to provide written informed consent for compassionate use
- Adequate organ function: differential blood count: hemoglobin ≥ 10 g/dl without transfusions, leukocyte count > 3000/µl; liver enzymes ≤ 2 x upper limit of normal (ULN); serum creatinine ≤ 1.5 mg/dl or calculate creatinine clearance ≥ 50 ml/min,

Exclusion criteria

- Contraindications for treatment with DMF (known hypersensibility to the drug, severe gastrointestinal disease (like ulcerations), Alcohol abuse, other obligately liver- or nephrotoxic medication, known clinically apparent renal or hepatic insufficiency, pregnancy), 8- Methoxy psoralene (idiosyncratic reactions to psoralenes, melanoma, aphakia), or extracorporeal photopheresis (inability to tolerate extracorporeal volume loss, leukocyte count > 25000/µl, thrombocyte count < 25000/µl, hemaoglobin < 8 g/dl, hematocrit < 27%, splemectomy, latent or floride infections, contraindications for full heparinization)
- Pregnant or lactating patients

Supplementary figures

Supplementary Figure 1

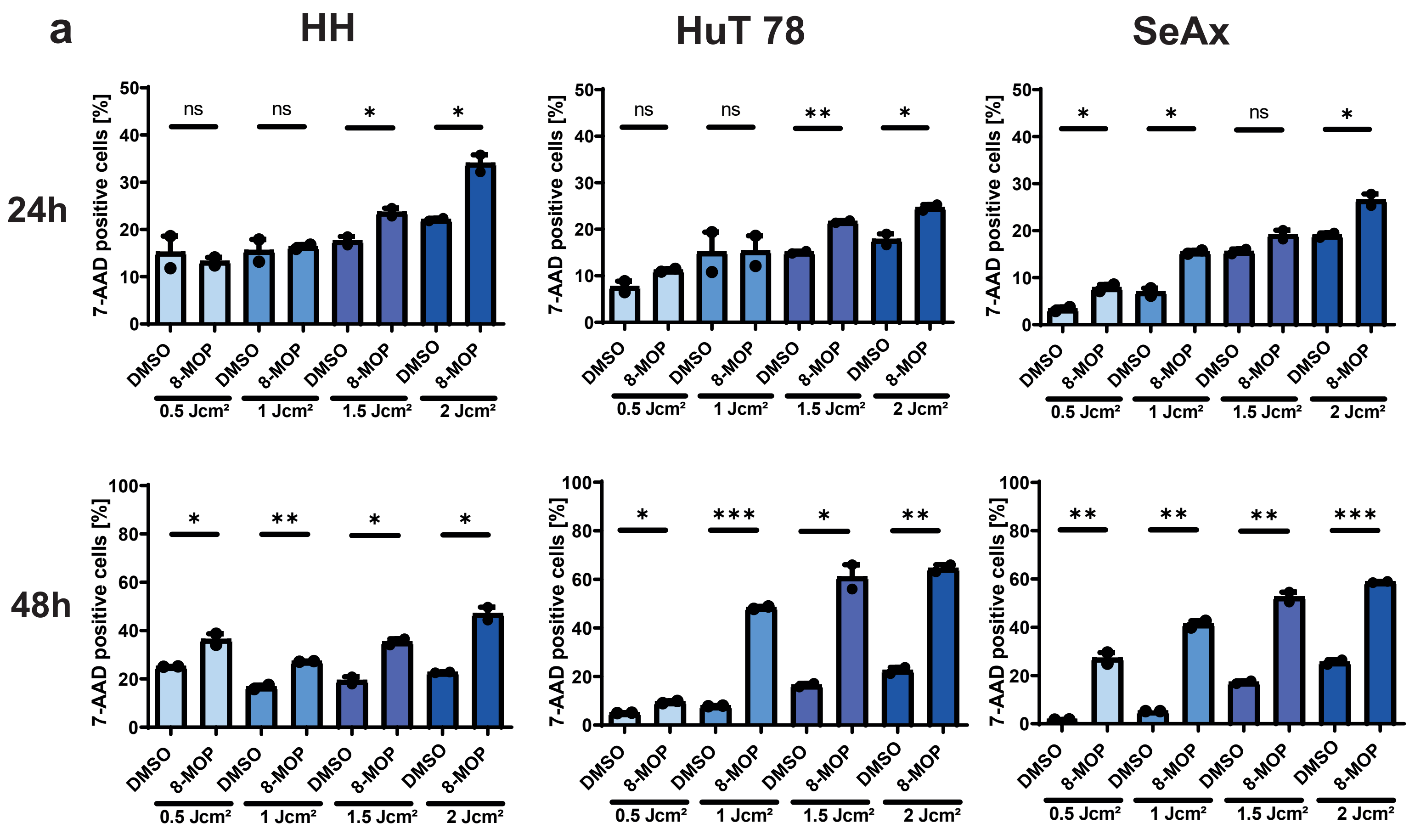

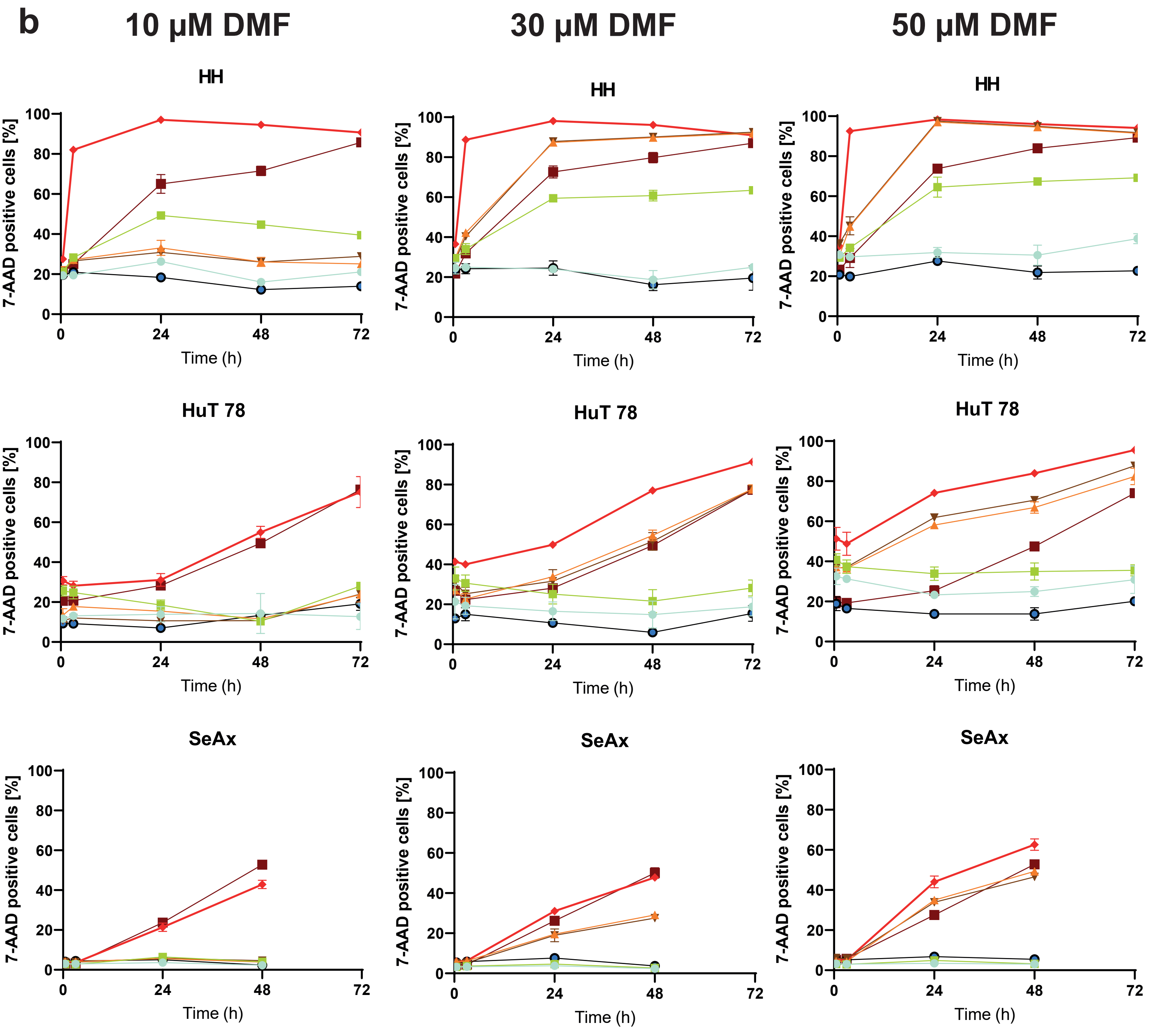

Titration assays. (a) Titration assay of the cell lines HH, Hut-78, and SeAx for UVA in the presence of 200 ng/ml 8-MOP and DMSO after 24 h and 48 h. (b) Titration curves for of the cell lines HH, Hut-78, and SeAx for DMF at concentrations of 10 µM, 30 µM, and 50 µM in the presence of 200 ng/ml 8-MOP and the pre-determined UVA dosage at 0 h, after 24 h, 48 h, and 72 h. Cell death was measured by 7AAD staining with FACS. The level of significance is indicated by asterisks (**** ≤ 0.0001; *** ≤ 0.001; ** ≤ 0.01; * ≤ 0.05). Error bars show the standard deviation.

Supplementary Figure 2

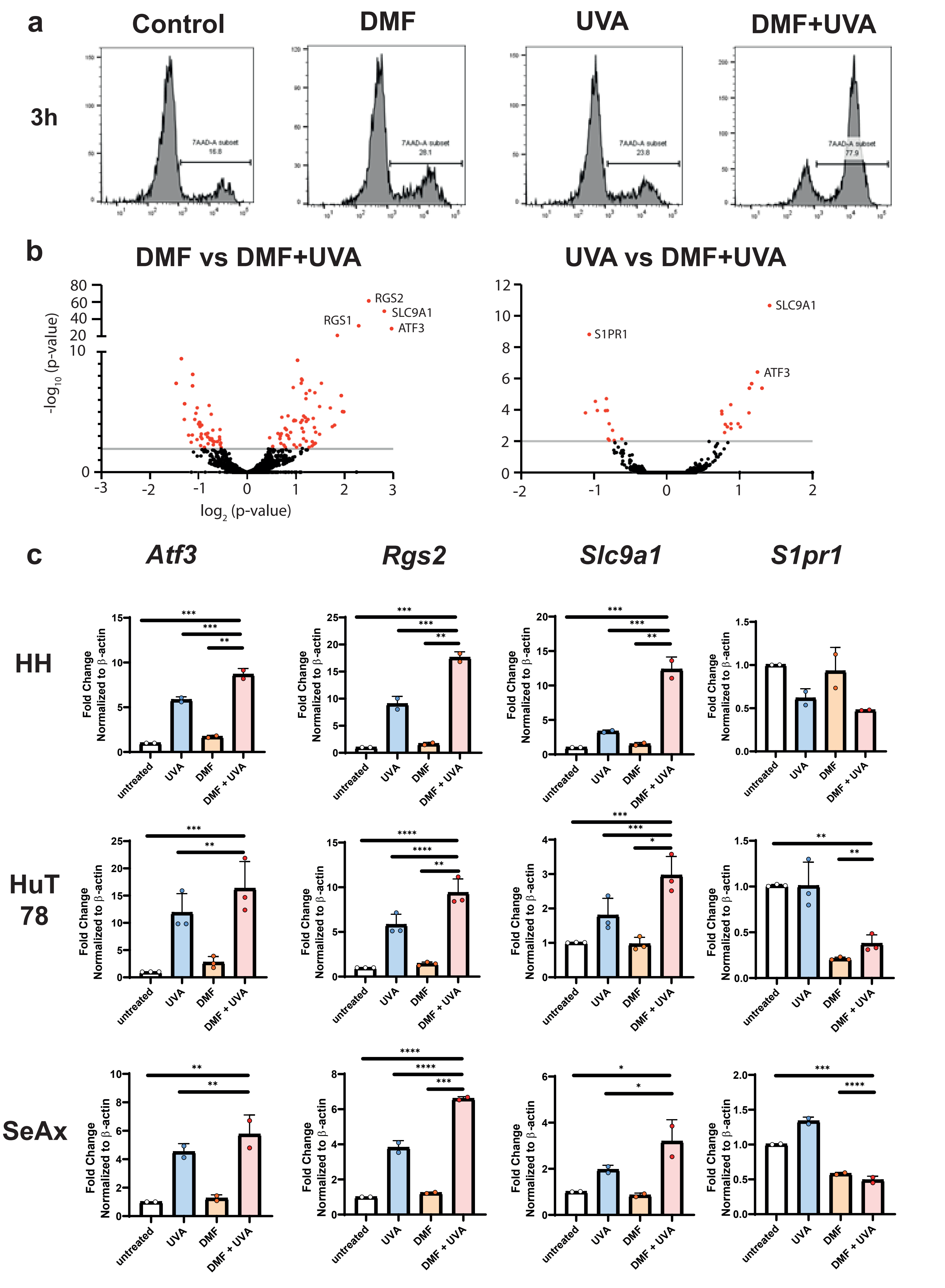

Transcriptomic analysis of CTCL cell lines upon DMF-8-MOP/UVA treatment. HH cells were reated with DMF monotreatment, 8-MOP/UVA monotreatment, and the combination treatment with DMF and 8-MOP/UVA, and compared to untreated controls. (a) Histogram of the 7-AAD positive cells detected by FACS after 3 h. All experiments were performed in triplicates. (b) Volcano plots of the differentially expressed genes of the HH cells treated with DMF, 8-MOP/UVA compared to the combination treatment. The significance threshold of $p < 0.01$ is indicated by the horizontal line. The genes with the highest significance are named in the graph. (c) HH, HuT 78 and SeAx cells were treated with DMF monotreatment, 8-MOP/UVA monotreatment, and the combination treatment with DMF and 8-MOP/UVA, and compared to untreated controls. The mRNA expression fold change (normalized to β-actin) was detected by qPCR for *Atf3*, *Rgs2*, *Slc9a1*, and *S1pr1*. All experiments were performed in triplicates. The level of significance is indicated by asterisks (**** ≤ 0.0001; *** ≤ 0.001; * * ≤ 0.01; * ≤ 0.05). Error bars show the standard deviation.

Supplementary Figure 3

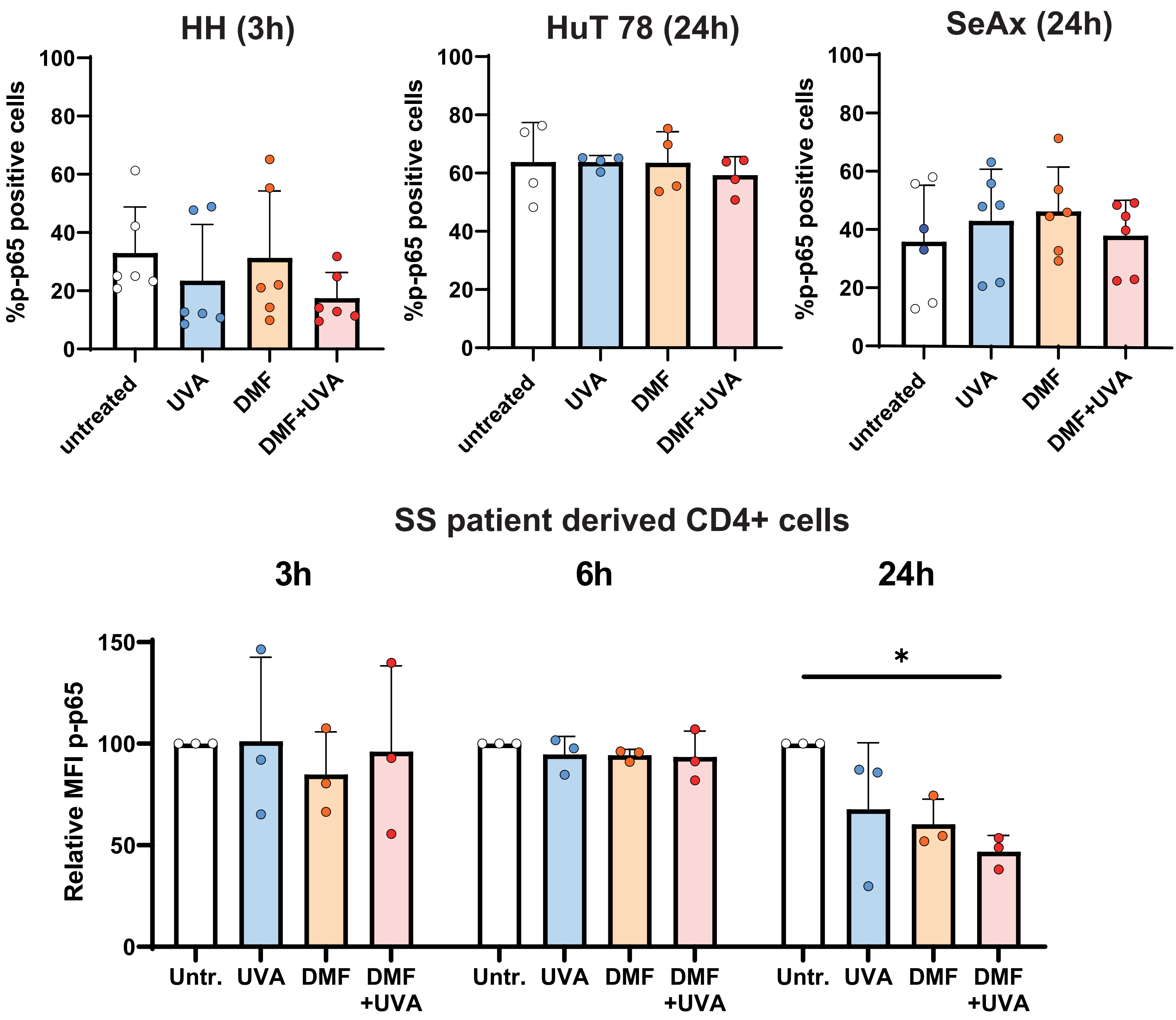

NF-κB p-p65 expression in CTCL cell lines and CD4+ SS patient cells (n=3). The cells were treated with DMF monotreatment, 8-MOP/UVA monotreatment, and the combination treatment with DMF and 8-MOP/UVA, and compared to untreated controls. A p-p65 staining was performed and the amount p-p65 positive cells in % were measured by FACS after 3 h for HH cells, and after 24 h for Hut-78 and SeAx cells. All experiments were performed in triplicates. The level of significance is indicated by asterisks (**** ≤ 0.0001; *** ≤ 0.001; ** ≤ 0.01; * ≤ 0.05). Error bars show the standard deviation.

Supplementary Figure 4

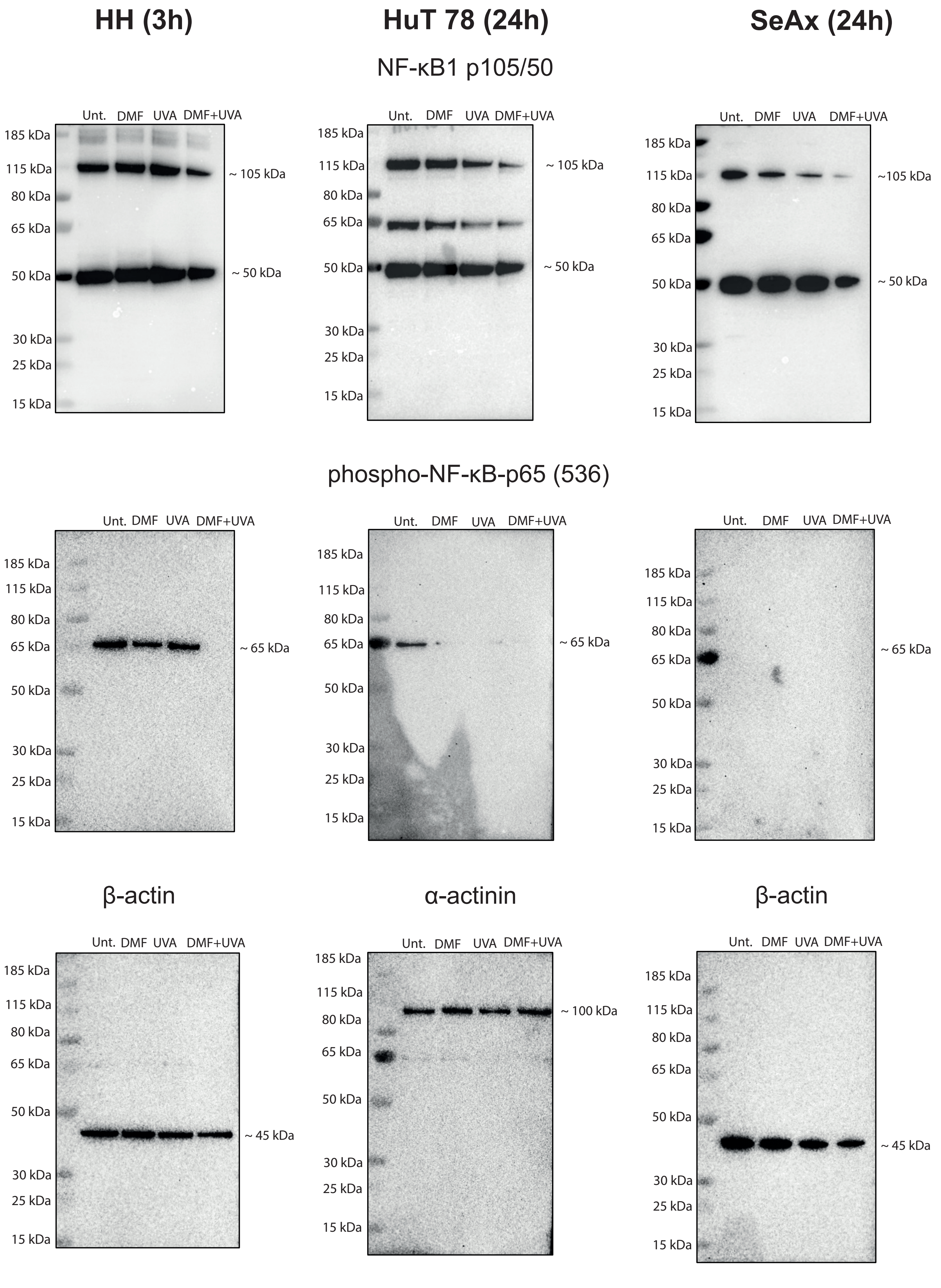

Immunoblot membranes of the immunoblots presented in Fig. 3b for the NF-κB1 p105/50, phospho-NF-κB-p65 (S536), β-actin, and α-actinin stainings.

Supplementary Figure 5

a

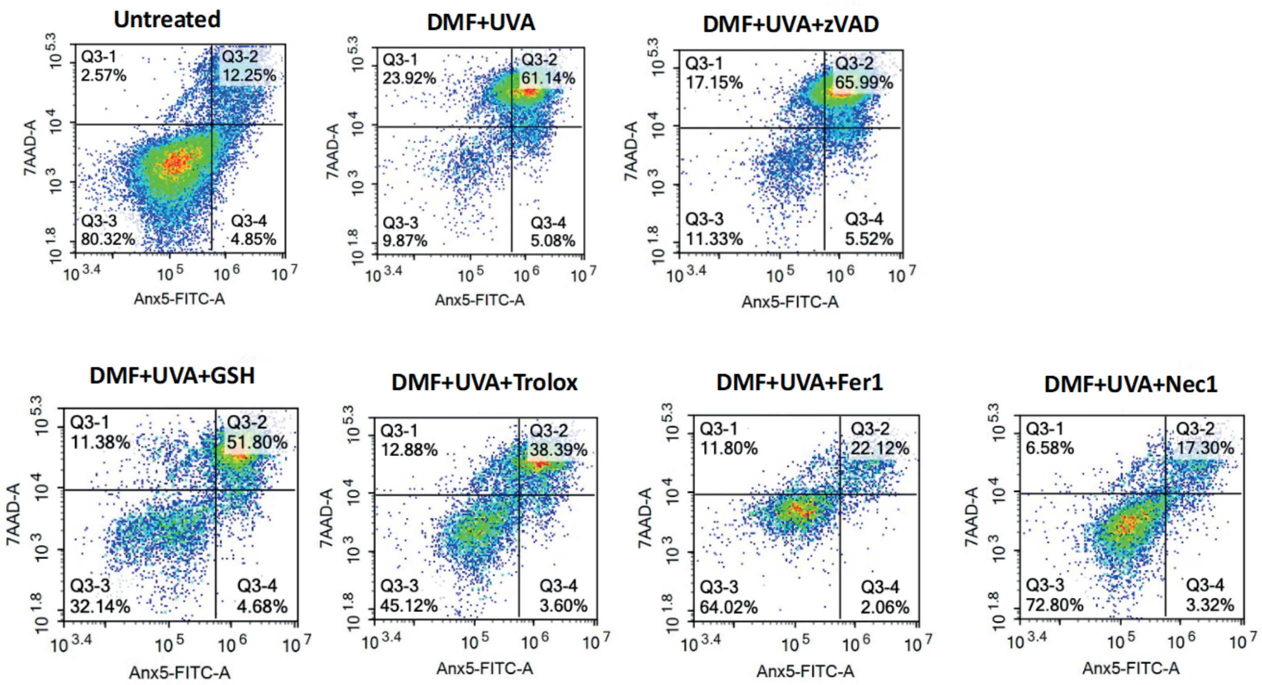

b

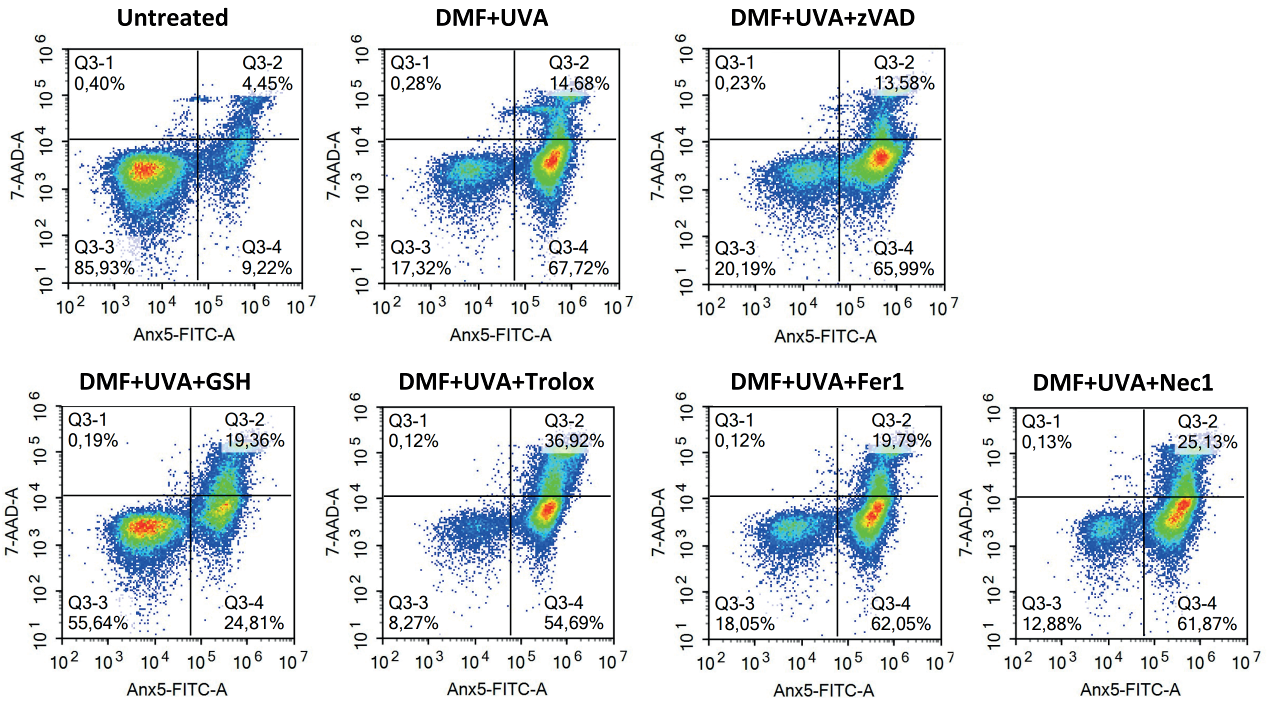

Representative FACS plots for the 7AAD/Annexin V staining in HH cells (a) and patient 9 for the DMF-8-MOP/UVA-combination and the respective inhibitors and the untreated controls.

Supplementary Figure 6

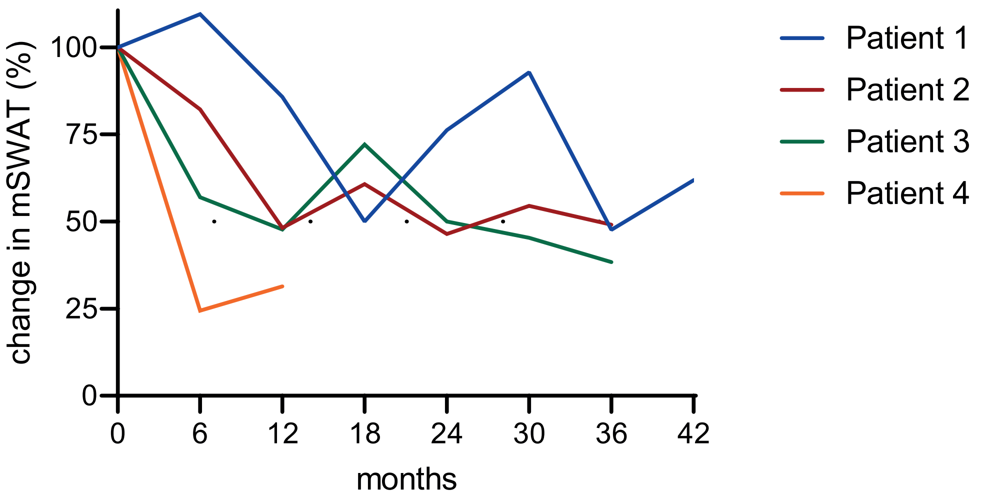

mSWAT change (in %) during the combination therapy of DMF and ECP of patient 1 (blue), patient 2 (red), patient 3 (green), and patient 4 (orange) over time (in months).

Supplementary tables

Supplementary Table 1 Conditions of the treatment of the CTCL cell lines and the patient-derived CTCL cells

Name	UVA (J/cm²)	DMF (µM)	MOP (ng/ml)
HH	2	30	200
Hut78	1	30	200
SeAx	1	50	200
Patient 2	0.5	50	400
Patient 3	0.5	50	400
Patient 4	1	50	400
Patient 5	0.75	50	400
Patient 6	0.5	50	400
Patient 7	0.5	30	400
Patient 8	0.5	30	400
Patient 9	0.5	30	400

Supplementary Table 2 Antibodies

antibody	cat. no.	manufacturer	application
NF-κB1 p105/p50	3035	Cell Signaling Technology, Cambridge, UK	Immunoblot
phospho-NF-κB p65	3033	Cell Signaling Technology, Cambridge, UK	Immunoblot
β-actin	5125S	Cell Signaling Technology, Cambridge, UK	Immunoblot
α-actinin	sc-17829	Santa Cruz Biotechnology, Dallas, Texas, USA	Immunoblot
CD4 (host rabbit)	ab133616	abcam, Cambridge, UK	Immunofluorescence
CD4 (host mouse)	M7310	Agilent, Santa Clara, California, USA	Immunofluorescence
CD163	MA5-11485	Thermo Fisher, Waltham, Massachusetts, USA	Immunofluorescence
CD8	ab4055	abcam, Cambridge, UK	Immunofluorescence
CD56	ab75813	abcam, Cambridge, UK	Immunofluorescence

Supplementary Table 3 Patient cohort

**Patient**	**gender, age**	**TNM stage**	**Leuko-cytes/nl**	**T lympho-cytes [%]**	**CD4/µl**	**SC/µl**	**CD4/ CD8**	**mSWAT**	**treatment**
1	m, 73	T4NxM0B2	18.04	93.8	10070	9810	44.3	42	IFN, ECP, DMF, Bexa, PUVA
2	m, 67	T4N1M0B2	9.74	95.2	2803	2525	495.5	112	ECP, DMF, Bexa, MTX, PUVA
3	f, 76	T4N3bM0B2	5.31	60.4	974	419	2.0	86	ECP, DMF, IFN, TSEB
4	m, 73	T4NxM0B2	11.47	86.6	3766	3011	13.7	86	ECP, IFN, MTX
5	w, 80	T4N0M0B2	12.6	95.7	5099	4190	17.8	NA	ECP, Brent, Moga, DMF, TSEB, IFN, Bexa
6	m, 73	T4N3M1B2	21.07	91.3	8567	4266	12.7	NA	PUVA, IFN, Bex, DMF, ECP, MTX, Brent, Gem
7	f, 75	T4NxM0B2	10.49	93.1	3139	993	25.7	NA	ECP, Moga, DMF, IFN, PUVA
8	m, 58	T4NxM0B2	10.77	91.7	3047	2544	10.9	NA	ECP, IFN
9	m, 80	T4N2M0B2	21.7	95.5	11692	1036	94.5	NA	Bex, ECP, IFN
Norm			4.2–10.2	60-83	528-1495	<1000	1–2.8		

Clinical data of the human patient collective of SS patients. Bex = Bexarotene; Brent = Brentuximab; DMF = Dimethyl fumarate; ECP = Extracorporeal Photopheresis; Gem = Gemcitabine; IFN = Interferon; Moga = Mogamulizumab; MTX = Methotrexate; PUVA = Psoralene plus ultraviolet A radiation; SC = Sézary cells; TSEB = Total skin electron beam therapy.

Supplementary Table 4 Treatment course of the four patients with the DMF/ECP combination treatment since the first introduction of the ECP in the therapeutic regimen

Months	Therapy
**Patient 1**	
0 - 5	ECP/IFN-combination therapy
6 - 13	DMF monotherapy
14 - 18	ECP/IFN-combination therapy
19 - 58	ECP/IFN/DMF-combination therapy
59 - 62	Monotherapy with Mogamulizumab
63 - 67	Monotherapy with Brentuximab vedotin
68 - 73	Monotherapy with ECP
74 - 80	ECP/DMF-combination therapy
**Patient 2**	
0 - 10	ECP monotherapy
11 - 15	DMF monotherapy
16 - 35	ECP/DMF-combination therapy
36 - 73	ECP maintenance therapy
**Patient 3**	
0 - 5	ECP monotherapy
6 - 12	DMF monotherapy
13 - 22	ECP/DMF-combination therapy
23 - 30	ECP/IFN-combination therapy
31 - 41	ECP/IFN/DMF-combination therapy
42 - 66	ECP/DMF-combination therapy
**Patient 4**	
0 - 3	Combination therapy with ECP and IFN (1 administration)
4	Combination therapy with ECP and MTX
5 – 21	ECP/DMF-combination therapy (ongoing)

DMF = Dimethyl fumarate; ECP = Extracorporeal Photopheresis; IFN = Interferon; MTX = Methotrexate
